# Annoyance Caused by Noise and Air Pollution during Pregnancy: Associated Factors and Correlation with Outdoor NO_2_ and Benzene Estimations

**DOI:** 10.3390/ijerph120607044

**Published:** 2015-06-18

**Authors:** Ana Fernández-Somoano, Sabrina Llop, Inmaculada Aguilera, Ibon Tamayo-Uria, María Dolores Martínez, Maria Foraster, Ferran Ballester, Adonina Tardón

**Affiliations:** 1 Spanish Consortium for Research on Epidemiology and Public Health (CIBERESP), Madrid 28029, Spain; E-Mails: llop_sab@gva.es (S.L.); ibontama@gmail.com (I.T.-U.); ballester_fer@gva.es (F.B.); atardon@uniovi.es (A.T.); 2 Department of Preventive Medicine and Public Health, University of Oviedo, Asturias 33006, Spain; 3 Unit of Epidemiology and Environmental Health, FISABIO—Universitat Jaume I—Universitat de València Joint Research, Valencia 46020, Spain; 4 Swiss Tropical and Public Health Institute, Basel 4002, Switzerland; E-Mails: iaguilerajim@gmail.com (I.A.); maria.foraster@unibas.ch (M.F.); 5 University of Basel, Basel 4003, Switzerland; 6 Health Research Institute, Biodonostia, San Sebastián 20014, Spain; E-Mail: dml-martinez@ej-gv.es; 7 Department of Environment and Regional Planning, Basque Government, San Sebastian 20004, Spain; 8 Centre for Research in Environmental Epidemiology (CREAL), Barcelona 08003, Spain; 9 Hospital del Mar—Research Institute (IMIM), Barcelona 08003, Spain

**Keywords:** annoyance, benzene, environmental pollution, pregnancy, nitrogen dioxide, noise

## Abstract

This study aimed to describe the degree of annoyance among pregnant women in a Spanish cohort and to examine associations with proximity to traffic, NO_2_ and benzene exposure. We included 2457 participants from the Spanish Childhood and Environment study. Individual exposures to outdoor NO_2_ and benzene were estimated, temporally adjusted for pregnancy. Interviews about sociodemographic variables, noise and air pollution were carried out. Levels of annoyance were assessed using a scale from 0 (none) to 10 (strong and unbearable); a level of 8 to 10 was considered high. The reported prevalence of high annoyance levels from air pollution was 11.2% and 15.0% from noise; the two variables were moderately correlated (0.606). Significant correlations between NO_2_ and annoyance from air pollution (0.154) and that from noise (0.181) were observed. Annoyance owing to noise and air pollution had a low prevalence in our Spanish population compared with other European populations. Both factors were associated with proximity to traffic. In multivariate models, annoyance from air pollution was related to NO_2_, building age, and country of birth; annoyance from noise was only related to the first two. The health burden of these exposures can be increased by stress caused by the perception of pollution sources.

## 1. Introduction

There is strong epidemiological evidence that outdoor air pollution contributes to morbidity and mortality [1,2]. Noise is also associated with a variety of harmful health effects [3,4]. Although relative risks related to air pollution and noise are rather small, the public health impact is substantial owing to the ubiquitous exposure distribution in the population.

The scientific community has begun to pay more attention not only to the proven health hazards caused by factors such as air pollution and noise, but also to reactions that are important for human well-being and yet are rarely studied, including distress or annoyance in relation to sensory perception of harmful environmental agents, and possible subsequent adverse effects of a psychological (quality of life) as well as physiological (prenatal development) nature [5,6].

The World Health Organization defines annoyance as “a feeling of displeasure associated with any agent or condition, known or believed by an individual or group to adversely affect them” [7]. This definition implies an effect that may not be pathogenically demonstrable but that involves a negative factor for the individual’s comfort and well-being. Additionally, this definition could allude to the impact of air pollution and noise on health. Thus, air pollution and noise could also have psychological effects on individuals because these factors are perceived as a nuisance and environmental stressors that limit quality of life and well-being [8,9]. Nonetheless, the health effects of perceived annoyance from air pollution and noise remain poorly understood [10].

Recent evidence suggests potential stress-related modifications that are associated with a wide range of pollutant exposures on health outcomes. In this sense, stress, which can influence immune function and susceptibility to illness, may potentiate the effects of air pollution on respiratory disease development and exacerbation [5].

Stress can be detrimental at any age. However, there may be critical periods, such as during early immune development, when stress is particularly influential in shaping future susceptibility and disease risk [11]. With respect to noise, children represent a group that is particularly vulnerable to non-auditory health effects. Children have less cognitive capacity to anticipate and understand stressors and lack well-developed coping strategies [3].

Maternal stress during pregnancy may influence immune function and health in neonates [12]. In addition, parental stress has been used to examine stress exposure in young children [5]. Prospective studies have linked stress exposures during prenatal development with broad biological and psychological vulnerabilities, affecting neuroendocrine, immune, metabolic, and growth processes [5,13]. Although not entirely immutable, these effects may have permanent and compounding consequences, especially if not addressed early [5,9,14]. Annoyance, as a first negative reaction, could be an early warning of health impairment [15].

Distress caused by air pollution and noise is frequent; such annoyance has been recently reported in Europe [5,16,17,18,19,20,21]. It is important to assess several other characteristics that have a great impact on annoyance reporting [10,18,22,23] as stated in a previous study of one cohort that is included in the present work [24]. The fact that the association between exposure and annoyance is not always strong underscores the need for prospective studies to evaluate effects, and the necessity to explore the determinants of annoyance in each study area.

Information about the relationship between exposure to environmental stressors and annoyance responses is scarce [15]. Accurately characterizing both social and physical exposures is a notable challenge that must be undertaken carefully, especially in light of potential confounding across the exposures of interest.

The aims of the present study are to describe the degree of annoyance caused by air pollution and noise among women participants in a Spanish birth cohort study, to examine the correlation with estimated outdoor NO_2_ and benzene levels, and to study the factors associated with annoyance.

## 2. Methods

### 2.1. Study Population

The INMA (Childhood and Environment) study is a multicenter population-based mother and child cohort study conducted in different areas of Spain, following a common protocol [25]. Our study included 2457 pregnant women from the provinces of Asturias, Gipuzkoa and Valencia, and the city of Sabadell in Barcelona province. Pregnant women were enrolled from 2003 to 2008 during their first trimester of pregnancy at public primary health care centers or public hospitals, depending on the region and, provided they fulfilled the inclusion criteria (≥16 years of age, intention to deliver at the reference hospital, no problems with communication, singleton pregnancy, and no assisted conception). The hospital ethics committees of each region approved the research protocol and all women gave their written informed consent prior to inclusion in the study.

#### Assessment of Air Pollution Exposure

A complete description of the exposure modeling methods used has been reported previously [26,27,28,29,30]. Briefly, ambient concentrations of nitrogen dioxide (NO_2_) and benzene were measured repeatedly during participants’ pregnancy, with passive samplers distributed throughout the study areas. The samplers measured pollutant levels using radial symmetry (radiello^®^; Fundazione Salvatore Maugeri, Padua, Italy), with exposures during various sampling periods of 7 days each (Asturias: two sampling campaigns with 67 sampling points each; Gipuzkoa: two campaigns of 85 points; Sabadell: four campaigns of 57 points; Valencia: four campaigns of 93 points). Geographic information system data (land coverage, altitude, and distance to roads) derived in ArcGIS version 9.1 (ESRI, Redlands, CA, USA) were used to obtain predictor variables [31]. Land use regression (LUR) models with geographical information on land use, traffic, and altitude were used to predict NO_2_ and benzene levels at participants’ residential addresses. Residential changes during pregnancy were taking into account if the participant lived at least 2 months of the pregnancy period in a new residence, which occurred in 1%–6% of cases, depending on the cohort. Spatial estimates were temporally adjusted using serial records from the network of monitoring stations covering the study areas, to obtain estimates for each participant’s specific pregnancy period. Finally, an average exposure level over the entire pregnancy period was calculated.

### 2.2. Questionnaires

Annoyance scores were determined from questionnaires administered at 32 weeks’ gestation. Questions regarding air pollution and noise included: “To what extent does air pollution outside your home (gases, fumes, dust, and so on, from traffic, industry, and so on) annoy you if you leave the windows open?” and “To what extent does outdoor noise (from traffic, industry, and so on) annoy you inside your home if you leave the windows open?” Both degrees of annoyance were measured by an 11-point scale ranging from 0 (none) to 10 (strong and unbearable). We further defined categorical annoyance variables, considering high annoyance to be values of 8–10 on the original 11-point scale, medium annoyance as values of 4–7, and low annoyance values of 1–3 [23]. Participants who reported no annoyance (value 0) were considered the reference level in the analyses.

Questionnaires also included additional sociodemographic data: age, country of birth, education level, employment status, occupation; data on smoking, and housing data. We defined parental social class according to maternal or paternal occupation during pregnancy with the highest social class, according to a widely used Spanish adaptation of the International Standard Classification of Occupations (ISCO-88) coding system [32]. Additionally, information on traffic characteristics was collected. Thus, the questionnaire asked about the frequency of cars and heavy vehicles on the street nearest the home: (continuous (ref), fairly often, little, almost never) and the distance from the home to a street where traffic passes continuously, considering this in continuous and categorical: >1000 m (ref)/51–1000 m/≤50 m.

### 2.3. Statistical Analysis

The distribution and prevalence of high air pollution and noise annoyance were described. A bivariate analysis was performed to establish associations of individual variables with perceived annoyance. We used Mann–Whitney and Kruskal–Wallis tests for dichotomous and categorical variables respectively, and Spearman correlation for continuous variables. Finally, for multivariable analysis, we used ordinal logistic regression, with categorical variables of perceived annoyance as the dependent variable and socio-demographic and housing variables related to the outcome in bivariate analysis as independent variables. The models were built based on those variables with a significance level of *p* < 0.2 in the bivariate analysis, whereas variables with significance *p* < 0.1 were maintained in the model according to the likelihood ratio test. We decided to keep NO_2_ and benzene estimated levels as variables in the final model, even if they were not statistically significant. Finally, we performed sensitivity analyses to assess the robustness of our results. Thus, multivariable–adjusted models were further adjusted for socioeconomic variables that might act as confounders (education, working situation, social class, smoking, and age), to minimize the likelihood of residual confounding. However, none of these modified the coefficients more than 10% (percentage change 0%–3%); thus the decision was made not to include these variables in the models. Statistical analyses were performed using SPSS for Windows, Version 15.0 (SPSS Inc., Chicago, IL, USA) and Stata Statistical Software: Release 12.1 (StataCorp LP, College Station, TX, USA).

## 3. Results

**Figure 1:**
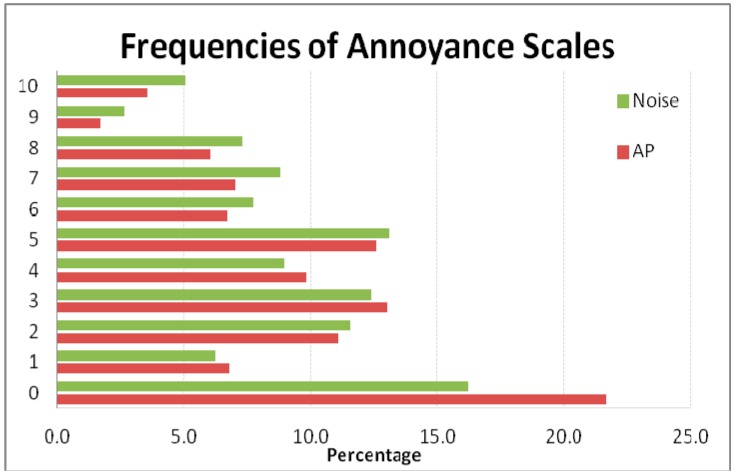
Distribution of air pollution and noise annoyance responses.

A total 11.2% of the study population reported high annoyance levels (8–10) owing to air pollution and 15.0% owing to noise (Table 1). The highest reported annoyance was in the Sabadell cohort both for air pollution (15.5%) and noise (21.2%). Additionally, 6.4% of all participants were highly annoyed by both air pollution and noise. Median scores were 3 for atmospheric and 4 for noise pollution. The complete distribution of the annoyance scales is shown in Figure 1.

**Table 1:** Prevalence for each category of annoyance.

	Total		Asturias		Gipuzkoa		Sabadell		Valencia		*p*-Value *
	*n*	%	*n*	%	*n*	%	*n*	%	*n*	%	
**AP Annoyance**											
No (0)	533	21.7	55	12.1	181	30.1	73	11.9	224	28.5	<0.001
Low (1–3)	759	30.9	194	42.5	156	26.0	179	29.2	230	29.3	
Medium (4–7)	889	36.2	161	35.3	193	32.1	267	43.5	268	34.1	
High (8–10)	276	**11.2**	46	**10.1**	71	**11.8**	95	**15.5**	64	**8.1**	
**Noise Annoyance**											
No	399	16.2	46	10.1	136	22.7	45	7.3	172	21.9	<0.001
Low	742	30.2	202	44.3	139	23.2	145	23.6	256	32.6	
Medium	947	38.6	155	34.0	245	40.8	294	47.9	253	32.2	
High	368	**15.0**	53	**11.6**	80	**13.3**	130	**21.2**	105	**13.4**	
Total	2457	100.0	456	100.0	601	100.0	614	100.0	786	100.0	

* Chi2.

Country of birth and residential building age were associated with air pollution and noise annoyance in the bivariate analysis. Foreign-born women and those living in older homes reported a higher degree of discomfort. Lower educational level and lower social class were also associated with air pollution annoyance (Table 2).

**Table 2:** Pregnant women characteristics and descriptives for annoyance levels.

	*n*	%	AP Annoyance						Noise Annoyance					
			Mean	S.D.	P_25_	P_50_	P_75_	*p* *	Mean	S.D.	P_25_	P_50_	P_75_	*p* *
**Cohort**														
Asturias	456	18.48	4	3	2	3	5	**<0.001**	4	3	2	3	6	**<0.001**
Gipuzkoa	592	23.96	3	3	0	3	5		4	3	1	4	6	
Sabadell	614	25.03	4	3	2	4	6		5	3	3	5	7	
Valencia	786	32.53	3	3	0	3	5		4	3	1	3	6	
**Age**														
<25	176	0.079	4	3	2	3	6	0.965	4	3	1	4	7	0.945
25–29	787	0.32	4	3	1	3	5		4	3	2	4	6	
30–34	1037	0.417	4	3	1	3	5		4	3	2	4	6	
35+	447	0.184	4	3	1	3	6		4	3	2	4	6	
**Country of origin**														
Spain	2.239	91.5%	4	3	1	3	5	**<0.001**	4	3	2	4	6	**0.002**
Other	202	8.5%	4	3	2	5	7		5	3	2	5	7	
**Education**														
Primary	597	25.1%	4	3	1	4	6	**0.023**	4	3	2	4	7	0.642
Secondary	1.014	41.4%	4	3	1	3	5		4	3	2	4	6	
University	833	33.4%	3	3	1	3	5		4	3	2	4	6	
**Working situation**														
Employed	1.896	77.3%	3	3	1	3	5	0.070	4	3	2	4	6	0.849
Unemployed	315	13.1%	4	3	1	4	6		4	3	2	4	7	
Student	25	1.0%	4	3	2	3	6		4	3	2	4	5	
Housewife	209	8.6%	4	3	2	4	6		4	3	1	4	6	
**Social Class**														
SC I+II	525	21.3%	3	3	1	3	5	**0.012**	4	3	2	4	7	0.292
SC III	634	25.7%	3	3	1	3	5		4	3	2	4	6	
SC IV+V	1.288	53.0%	4	3	1	4	6		4	3	2	4	6	
**Smoker**														
No	1.101	0.45	3.65	2.8	1	3	6		4.15	2.9	2	4	6	
Yes	1344	0.55	3.54	2.9	1	3	5		4.03	3	2	4	6	
**Age of the house**														
<5	613	25.7%	3	3	1	3	5	**<0.001**	4	3	1	3	6	**<0.001**
5–14	584	24.5%	4	3	1	3	6		4	3	2	4	6	
15–29	416	17.5%	4	3	1	4	6		4	3	2	4	7	
>29	768	32.3%	4	3	1	4	6		4	3	2	4	7	

* Kruskal-Wallis or Mann-Whitney test.

Moreover, we also found that there was an association between estimated NO_2_ exposure levels above the allowed threshold of 40 µg/m^3^ according to WHO air quality guidelines [33] and perceived high annoyance owing to air pollution and, even more so, to noise. No association with levels of exposure to benzene above the allowed threshold (5 µg/m^3^) was found because only 0.4% of the study population was exposed to such levels (Table 3).

**Table 3:** Distribution of annoyance levels by compliance of air pollution normative.

	NO_2_ > 40 µg/m^3^				*p* *	Benzene > 5 µg/m^3^				*p* *
	No		Yes			No		Yes		
	*n*	%	*n*	%		*n*	%	*n*	%	
**AP Annoyance**										
No	440	22.4	76	18.5	**0.002**	502	21.5	1	11.1	0.286
Low	621	31.6	110	26.8		717	30.7	1	11.1	
Medium	704	35.8	160	39.0		855	36.6	6	66.7	
High	201	10.2	64	15.6		262	11.2	1	11.1	
**Noise Annoyance**										
No	344	17.5	42	10.2	**<0.001**	378	16.2	1	11.1	0.951
Low	607	30.9	113	27.6		703	30.1	3	33.3	
Medium	753	38.3	161	39.3		901	38.6	4	44.4	
High	261	13.3	94	22.9		353	15.1	1	11.1	

* Chi2 test.

Spearman correlations of air pollution and noise annoyance with NO_2_ were low but positive and statistically significant. No association was found between benzene and noise annoyance or between benzene and atmospheric annoyance. Moderate and statistically significant correlations (range: |0.27|–|0.48|) of air pollution and noise annoyance with traffic-related variables were obtained. Those correlations were greater for noise (Table 4), and both annoyance scores were highly correlated (rho = 0.606).

Finally, according to ordinal multivariable logistic regression, NO_2_ and building age were the main determinants of noise annoyance, whereas these variables plus country of origin were the main predictors of air pollution annoyance. The models showed that the odds of reporting high air pollution annoyance were multiplied by 1.54 (95% CI: 1.39–1.71) for each 10 µg/m^3^ increase in NO_2_ exposure during pregnancy and by 1.73 (95% CI: 1.29–2.32) among foreign-born women. Furthermore, these odds were multiplied by 1.21 (95% CI: 0.98–1.50) if the residential building age was between 5 and 14 years (compared with buildings less than 5 years old), by 1.36 (95% CI: 1.08–0.72) if between 15 and 29 years, and by 1.33 (95% CI: 1.09–1.63) if the residence was 30 years old or more (Table 5).

As for noise annoyance, the odds of reporting high annoyance were multiplied by 1.70 (95% CI: 1.53–1.89) for each 10 µg/m^3^ increase in NO_2_ exposure during pregnancy; no association was found with benzene exposure. The odds of reporting higher annoyance was multiplied by 1.12 (95% CI: 0.90–1.38) if the building age was between 5 and 14 years (compared with buildings less than 5 years old), by 1.23 (95% CI: 0.97–1.56) if between 15 and 29 years, and by 1.28 (95% CI: 1.04–1.56) for residences 30 years old or greater (Table 5).

**Table 4:** Correlations (Spearman’s Rho) between annoyance reports and exposure and traffic related variables.

	AP Annoyance	Noise Annoyance	NO_2_ Pregnancy	Benzene Pregnancy	Car Frequency	Heavy Vehicles Freq	Distance to Traffic Street	Distance (Categories)
**AP Annoyance**	1	0.606 *	0.154 *	0.025	−0.342 *	−0.338 *	−0.330 *	0.268 *
**Noise Annoyance**		1	0.181 *	−0.010	−0.482 *	−0.445 *	−0.403 *	0.349 *
**NO_2_ pregnancy**			1	0.488 *	−0.114 *	−0.034	−0.126 *	0.108 *
**Benzene pregnancy**				1	−0.038	0.034	−0.131 *	0.121 *
**Car frequency ^a^**					1	0.634 *	0.544 *	−0.447 *
**Heavy vehicles frequency ^b^**						1	0.422 *	−0.333 *
**Distance to a traffic street ^c^**							1	−0.861 *
**Distance (categories) ^d^**								1

* *p* < 0.01. **^a^** Categories: continuous (ref)/fairly often/little/almost never; **^b^** Categories: continuous (ref)/fairly often/little/almost never; **^c^** Continuous; **^d^** Categories: >1000 m (ref)/51–1000 m/≤50 m.

**Table 5:** Multivariable ordinal logistic model for perceived annoyance and associated variables.

AP Annoyance	Coef	Cumulative OR	SE	Wald	df	*p*-Value	95%CI (Coef)		95%CI (Cum OR)	
NO_2_ pregnancy	0.04	1.54 ^d^	0.00	43.51	1	0.000	0.02	0.03	1.19 ^d^	1.37 ^d^
Benzene pregnancy	0.05	1.05	0.05	3.33	1	0.068	−0.18	0.01	0.84	1.01
Country of origin ^a^	0.55	1.73	0.15	10.28	1	0.001	0.19	0.77	1.20	2.16
Building age ^b^ 5–14	0.19	1.21	0.11	4.51	1	0.034	0.02	0.44	1.02	1.56
Building age 15–29	0.31	1.36	0.12	11.27	1	0.001	0.17	0.63	1.18	1.88
Building age > 29	0.29	1.33	0.10	19.47	1	0.000	0.25	0.65	1.28	1.92
Cohort ^c^ = Gipuzkoa	−0.27	0.76	0.14	3.52	1	0.061	−0.55	0.01	0.57	1.01
Cohort = Sabadell	−0.13	0.87	0.18	0.55	1	0.458	−0.49	0.22	0.61	1.25
Cohort = Valencia	−1.24	0.29	0.15	71.96	1	0.000	−1.53	−0.95	0.22	0.39
**Noise Annoyance**										
NO_2_ pregnancy	0.05	1.70 ^d^	0.01	94.78	1	0.000	0.04	0.06	1.53 ^d^	1.89 ^d^
Benzene pregnancy	0.00	1.00	0.07	0.00	1	0.992	−0.14	0.14	0.87	1.15
Building age 5–14	0.11	1.12	0.11	1.00	1	0.318	−0.11	0.32	0.90	1.38
Building age 15–29	0.21	1.23	0.12	2.98	1	0.084	−0.03	0.44	0.97	1.56
Building age > 29	0.24	1.28	0.10	5.53	1	0.019	0.04	0.45	1.04	1.56
Cohort ^c^ = Gipuzkoa	0.12	1.12	0.14	0.66	1	0.417	−0.17	0.40	0.85	1.49
Cohort = Sabadell	0.14	1.15	0.18	0.59	1	0.441	−0.22	0.50	0.81	1.64
Cohort = Valencia	−1.13	0.32	0.15	59.77	1	0.000	−1.41	−0.84	0.24	0.43

^a^ Reference category: Spain; ^b^ Reference category: <5; ^c^ Reference category: Asturias; ^d^ For each 10 µg/m^3^ increase.

## 4. Discussion

More than half of our study subjects reported medium or high annoyance levels owing to noise, and almost half reported these levels owing to air pollution. Reported annoyance was higher in Sabadell, which is the only cohort located exclusively in an urban area. Our results suggest a relationship between perception (annoyance) of NO_2_ pollution and exposure (individual estimated levels). Therefore, more pollution means more perception of pollution. However, the correlation was weak in our results. In terms of participant socio-demographic characteristics, only country of origin and residential building age were related to the degree of perceived discomfort from air pollution; only building age was related to noise annoyance. Otherwise, annoyance from air pollution and noise were related to the proximity of their residences to traffic and the frequency of vehicles near their homes.

Compared with other European studies evaluating similar data with the same 11-point scale, the prevalence of high annoyance owing to air pollution found here (11.2%) was lower than the 18.1% found in the SAPALDIA in Switzerland [23] and the average 14% reported in 25 centers across 12 European countries included in the ECRHS II study [22]. However, the prevalence was clearly higher than the percentage reported by Rotko *et al.* for the five European cities in the EXPOLIS study (2.7%–7.1%), in which the score range for the high category of annoyance was 7–10 in these cities, but for Prague, where the prevalence of high annoyance level was 25.3%. If we were to consider values of 7–10 as high annoyance, the percentage in our study would be 18.3% [10]. The mean value of annoyance caused by air pollution (3.6) was consistently higher in our study compared with the other studies (3.2 in SAPALDIA, 2.2 in ECRHS II, and 1.4–2.0 in EXPOLIS, except for Prague where a mean of 4.3 was reported).

There are few studies reporting the prevalence of noise annoyance, but we found lower percentages of participants with high discomfort levels from noise than in other European (27.9%–35.8%) [20] or American (32%–51%) [17] studies. However, it should be borne in mind that in the European study, a questionnaire item referring to road traffic nuisance was used to create the annoyance variable; in the American study, the degree of discomfort was not queried but instead the population at risk of discomfort was estimated from exposure to noise levels. Therefore, it is very difficult to compare these measures of discomfort among the various geographic regions.

The rather high correlation found here between air pollution and noise annoyance is in agreement with other international studies [23], as is the low correlation found between annoyance scores and individual estimations of air pollution exposure [10,23,34,35].

We found an association between perceived annoyance from air pollution and socio economic status, as shown in previous studies [10,22,23,24,36]. Nonetheless, here the associations were weak and were not included in the final models. This suggests that residence location and age had more influence on the perception of discomfort rather than the specific characteristics of study participants. The age of the residence would reflect the theoretical degree of housing insulation. In our study, lower socioeconomic status, lower education and younger age corresponded to older housing. Moreover, the results show that participants who live in less insulated buildings are more bothered by air pollution and noise.

We also found an association between air pollution annoyance and participants’ country of origin. This could indicate that the relationship between exposure and annoyance may depend on the study area and social acceptance of local environmental conditions. This is in line with higher reported air pollution annoyance in other countries compared with Spain [22,23].

The fact that we found an association with NO_2_ (specific to traffic emissions) suggests that participants detected or were distressed by environmental pollution associated with traffic, which is consistent with findings reported in other European studies [19,20].

A major limitation of our study is that we did not include a measure of noise so we were unable to evaluate its association with the corresponding annoyance reports, as other studies with modeled layers of noise have done [17,20]. However, proximity to traffic (the main source of both air pollution and noise) was assessed. Furthermore, we found an association between noise and NO_2_ exposure, also coming from traffic, suggesting that noise annoyance is also a function of traffic noise. Other geographic measures of potential for noise, such as distance to certain establishments or amount of green-space would help us to estimate an individual measurement of noise exposure. Noise exposure assessment will be very useful in future works and will allow us to characterize the complex interaction between noise, annoyance and health.

By contrast, this work has several strengths such as use of LUR models, which has high predictive ability, to assess individual exposure to air pollutants [31,37,38,39,40]. A further strength is the use of a scale for collecting annoyance responses, which is superior to the use of open-ended questions [41] and is comparable with the literature, which we have evaluated at different sites and with different pollutants. Above all is the strength of the study design. For investigations of annoyance, prospective studies are likely to provide superior data because cohort studies allow for better control of confounding factors.

Contrary to the argument of some authors that discomfort is a good indicator of exposure [22,42], the results of this study suggest that perception is a significant predictor of well-being, in the keeping with the findings of Atari *et al.* [43]. As stated earlier, our results also highlight the importance of verifying these relationships in all study areas, because they may vary between regions.

It is important to collect information about the relationship between environmental pollution and annoyance, not only because this can be considered an indicator of traffic air pollution or long term exposure [23] but also because it takes into account the possible psychological (quality of life and well-being) and physiological (prenatal development) stress-related effects that may be caused by such discomfort. We must also keep in mind that these effects may act as effect modifiers on health problems of environmental origin [44,45]. For this purpose, correlations between the measure of annoyance and the exposure variable need not be high, but should consistently be in the anticipated direction, as in the present study.

We also found that high levels of annoyance were reported even when the estimated air pollution levels complied with current WHO guidelines [7,33,46,47]. This is in accordance with other air pollution-related effects, which seem to appear even at lower pollution levels than those established by the guidelines, emphasizing the need to reduce air pollution even further. Therefore, air pollution sensitivity partly reflects a general environmental sensitivity but is also influenced by several individual factors, such as personality traits, living conditions and attitudes toward the pollutant source [23,35,36,48,49]. The influence of prior exposure to environmental stressors is another possible modifier of annoyance.

Our findings suggest that reported annoyance is a function of true exposure in the INMA population, though it is also a function of subjective factors which seem to vary across populations.

The combined effect of exposure to air pollution and noise, as well as the annoyance from both, can have health consequences, both directly from exposure [12,13,39,50] and indirectly from stress and psychological distress [6,23,30,36,51], including preterm delivery and deficient prenatal development of the respiratory or neuroendocrine system. This implies public health concerns and highlights the need to implement effective environmental policies [23,52] and technical risk assessment [43].

## 5. Conclusions

In this study, annoyance from air pollution was associated with the proximity of housing to traffic, residential building age and participants’ country of origin; noise annoyance was only related to the first two factors. Likewise, annoyance was associated with estimation of individual exposure to NO_2_, a pollutant related to traffic. The health burden of these exposures can be increased by stress caused by the perception of harmful environmental sources.
